# Supplementary material for: Radiomics Analysis of Whole-Kidney Non-Contrast CT for Early Identification of Chronic Kidney Disease Stages 1–3
Source: Bioengineering (Basel). 2025 Apr 25;12(5):454. doi: 10.3390/bioengineering12050454 (PMC12109281; doi:10.3390/bioengineering12050454)
Supplement: Supplementary file 1 [file bioengineering-12-00454-s001.zip › bioengineering-3578819-supplementary.pdf]

Supplementary

Table 1: Inter-rater reliability assessment between attending radiologists using Cohen's kappa coefficient

data	Doctor2	Doctor1			Kappa	Standard Error	95% CI
		0	1				
All data	0	1803	15	1818 (82.7%)	**0.761**	0.020	0.723 - 0.799
	1	119	261	380 (17.3%)			
		1922(87.4%)	276(12.6%)	2198			
Training sets	0	1443	13	1456 (82.8%)	**0.761**	0.022	0.718 - 0.804
	1	94	208	302 (17.2%)			
		1537(87.4%)	221(12.6%)	1758			
Testing sets	0	360	2	362 (82.3%)	**0.762**	0.043	0.677 - 0.847
	1	25	53	78 (17.7%)			
		385(87.5%)	55(12.5%)	440			

Table 2. Performance metrics comparison across machine learning models.

MLmodel	cutoff	AUC (95%CI)		sensitivity		specificity		accuracy		precision		f1Score	
		Training	Testing	Training	Testing	Training	Testing	Training	Testing	Training	Testing	Training	Testing
GP	0.500	0.849(0.831-0.866)	0.790(0.748-0.831)	0.750	0.709	0.770	0.732	0.760	0.720	0.765	0.726	0.757	0.717
DT	0.500	0.769(0.748-0.791)	0.719(0.673-0.767)	0.635	0.618	0.747	0.722	0.691	0.671	0.715	0.690	0,672	0.652
Logistic	0.500	0.781(0.76-0.802)	0.741(0.695-0.787)	0.719	0.686	0.675	0.65	0.697	0.668	0.689	0.662	0.703	0.674
RF	0.500	0.747(0.725-0.769)	0.725(0.679-0.771)	0.735	0.705	0.618	0.609	0.676	0.657	0.658	0.643	0.694	0.673
SVM	0.500	0.789(0.769-0.81)	0.769(0.725-0.812)	0.722	0.7	0.687	0.677	0.705	0.689	0.698	0.684	0.710	0.692

ML，Machine learning；GP, Gaussian Process; DT, Decision Tree; RF, Random Forest; SVM，Support Vector Machine.

Table 3. Selected top-performing radiomic features and corresponding LASSO coefficients.

Feature name	LASSO coefficient
wavelet_glcm_wavelet-LLH-Contrast	0.148599967
discretegaussian_glszm_GrayLevelNonUniformity	0.071070194
wavelet_glcm_wavelet-HLH-Imc2	0.061660506
wavelet_glcm_wavelet-HHL-Correlation	0.033637002
wavelet_gldm_wavelet-LLH-SmallDependenceEmphasis	0.032024026
wavelet_glcm_wavelet-LLH-Correlation	0.028394666
wavelet_glcm_wavelet-LHL-Correlation	0.023129309
wavelet_glszm_wavelet-LLH-SizeZoneNonUniformityNormalized	0.023074336
wavelet_firstorder_wavelet-LLH-TotalEnergy	0.022420669
wavelet_glszm_wavelet-LLH-GrayLevelNonUniformityNormalized	0.017822871
wavelet_glrlm_wavelet-LLH-GrayLevelVariance	0.017127322
wavelet_glszm_wavelet-LLH-ZoneEntropy	0.002401254
wavelet_glszm_wavelet-HHL-GrayLevelNonUniformityNormalized	-0.013806593
wavelet_glszm_wavelet-LLH-ZonePercentage	-0.034051027
wavelet_glcm_wavelet-HLL-Imc2	-0.034960453
wavelet_glcm_wavelet-HLH-Imc1	-0.05639576
wavelet_glcm_wavelet-HHL-Imc2	-0.05664781
boxsigmaimage_glszm_SizeZoneNonUniformityNormalized	-0.057431825
wavelet_gldm_wavelet-LLH-DependenceNonUniformityNormalized	-0.07698523
wavelet_glrlm_wavelet-HLH-RunEntropy	-0.09967952
wavelet_gldm_wavelet-LLH-LargeDependenceEmphasis	-0.100514285
specklenoise_glcm_InverseVariance	-0.109387688
wavelet_firstorder_wavelet-LLH-90Percentile	-0.124509148
wavelet_gldm_wavelet-LLH-DependenceEntropy	-0.1256971
